# The quantitative comparison between high wall shear stress and high strain in the formation of paraclinoid aneurysms

**DOI:** 10.1038/s41598-021-87126-w

**Published:** 2021-04-12

**Authors:** Jung-Jae Kim, Hyeondong Yang, Yong Bae Kim, Je Hoon Oh, Kwang-Chun Cho

**Affiliations:** 1 Department of Neurosurgery, College of Medicine, Ewha Womans University, Ewha Womans University Seoul Hospital, Seoul, Korea; 2 Department of Mechanical Engineering and BK21 FOUR ERICA-ACE Center, Hanyang University, 55 Hanyangdaehak-ro, Sangnok-gu, Ansan, 15588 Gyeonggi-do Korea; 3 Department of Neurosurgery, College of Medicine, Yonsei University, Severance Hospital, Seoul, Korea; 4 Department of Neurosurgery, College of Medicine, Catholic Kwandong University, International St. Mary’s Hospital, Simgokro 100gil 25, Seo-gu, Incheon, Korea

**Keywords:** Anatomy, Computational neuroscience, Mechanical engineering, Predictive markers

## Abstract

In the hemodynamic study, computational fluid dynamics (CFD) analysis has shown that high wall shear stress (WSS) is an important parameter in cerebral aneurysm formation. However, CFD analysis is not more realistic than fluid–structure interaction (FSI) analysis given its lack of considering the involvement of vascular structures. To investigate the relationship between the hemodynamic parameters and the aneurysm formation, the locations of high WSS and high strain were extracted from the CFD and FSI analyses, respectively. Then the distances between the aneurysm formation site and the locations of high WSS or high strain were calculated. A total of 37 intracranial paraclinoid aneurysms were enrolled for quantitative comparison. Additionally, the dura mater was modeled to facilitate realistic results in FSI analysis. The average distance from the location of the aneurysm formation site to the high strain (1.74 mm $\pm$ 1.04 mm) was smaller than the average distance to the high WSS (3.33 mm $\pm$ 1.18 mm). The presence of dura mater also influenced the findings in the aneurysm formation site. High strain extracted by FSI analysis is an important hemodynamic factor related to the formation of cerebral aneurysms. Strain parameter could help to predict the formation of aneurysms and elucidate the appropriate treatment.

## Introduction

To develop new preventive and therapeutic strategies for intracranial aneurysms, it is crucial to understand the mechanisms of aneurysm formation^1^. Accordingly, many studies investigating the mechanisms of aneurysm formation have been conducted in the last several decades. Especially, hemodynamic parameters play a key role in aneurysm formation, growth, and rupture^2,3^.

Among the various hemodynamic parameters, wall shear stress (WSS) has been reported to be a predominant parameter influencing aneurysm formation due to relationship between high WSS and endothelial cells^4,5^.

Simultaneously with the increasing importance of WSS, computational fluid dynamics (CFD) has become widely used to reveal the impact of WSS on the aneurysm formation site^6,7^. Numerous studies have adopted CFD to investigate correlations between high WSS and the site of aneurysm formation^8–11^. However, CFD has a critical limitation in that it cannot consider the variation in blood vessels caused by blood flow and the effects of the structure surrounding blood vessels such as the dura mater since a rigid vessel wall is assumed in the CFD analysis^12^. The dura mater is a thick membrane that surrounds the brain and spinal cord within the skull, and it blocks cerebrospinal fluid leak within the brain. The dura mater is tightly attached to the vessel walls in the paraclinoid segment where the vessel enters the intracranial region; therefore, the dura mater affects the deformation and movement of the blood vessel near the paraclinoid segment. However, since CFD analysis assumes a rigid blood vessel wall, the structural interaction between the blood vessel and the dura mater cannot be considered.

To overcome the limitations of CFD, we utilized fluid–structural interaction (FSI) to investigate the effects of the deformation of blood vessels and the influence of the dura mater. Because FSI enables to consider structural variations of the blood vessels due to blood pressure and the interaction between blood vessels and dura mater^13^, it could yield more realistic results than CFD.

We investigated the relationship between the aneurysm formation and the values of WSS or strain. For quantitative analysis, the distances from the location of the aneurysm formation site to the points of high WSS or high strain were calculated from CFD and FSI results and statistically compared.

## Methods

### Data acquisition

The protocols used in this study were approved and the need for patient informed consent was waived by our Institutional Review Board (Catholic Kwandong University, International St. Mary’s Hospital). This study analyzed a total of 37 unruptured paraclinoid aneurysms in 36 patients. For this study, a paraclinoid aneurysm was defined as an aneurysm arising from the segment of the internal carotid artery (ICA) between the distal dural ring and the origin of the posterior communicating artery. Our patients’ mean age was 56.6 years (range 27–81 years) and the mean aneurysm size was 5.32 mm (range 1.78–11.99 mm). All data were obtained retrospectively from patients diagnosed via digital subtraction angiography from March 2018 to March 2020 at our institution. Ruptured aneurysms were excluded because they were difficult to obtain prior shape data and the shape was unclear.

### Elimination of the aneurysms

**Figure 1:**
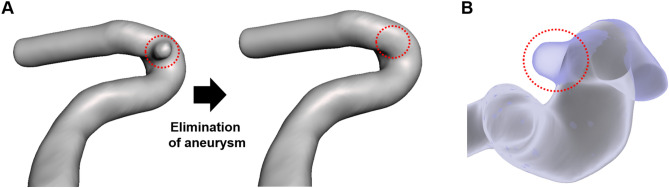
Example of the modified 3-dimensional blood vessel. The blood vessel model before and after aneurysm removal (**A**). The side view of overlapping model with and without aneurysm (**B**).

Aneurysms were manually removed to evaluate the results in the aneurysm formation site using commercial the computer-aided design programs CATIA (V5-6R2012; Dassault Systems, Paris, France) and Meshmixer (version 11.0.544; Autodesk, San Rafael, CA, USA). Figure 1A shows the models before and after an aneurysm was removed. When the two models overlapped, it could be confirmed that the aneurysm was reasonably removed (Fig. 1B).

### CFD and FSI analysis

CFD was conducted to calculate the WSS in an aneurysm formation site. The blood was assumed to be an incompressible Newtonian fluid with a density of 1,055 kg/m^3^ and a viscosity of 0.004 kg/m·s^14^. Also, pulsatile flow with a Womersley velocity profile was used in the inlet condition^15^. Flow waveform and flow rate were referenced in the study of Kono et al^16^. The time-averaged flow rate was 200 mL/min. Because the diameter of blood vessel differs from each patient, it was considered in generating a Womersley profile. MATLAB software (R2019b, Mathworks, USA) was used to calculate the Womersley profile. For the outlet condition, the pressure profile adjusted from the carotid artery was applied^17^. In the CFD analysis, the blood vessel was modeled as a rigid wall with a nonslip condition. CFD was performed using ANSYS Workbench Fluent (version 19.2; ANSYS Inc., Canonsburg, PA, USA).

**Figure 2:**
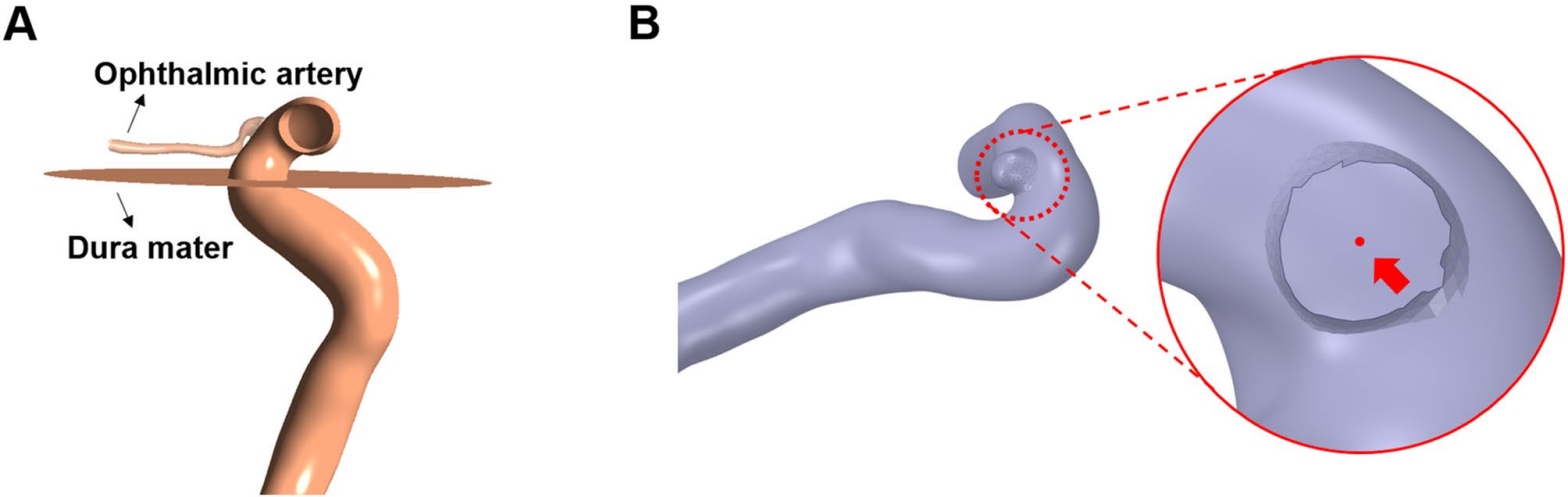
The methods determining the locations of the dura mater and the aneurysm formation site. The location of the dura mater was assumed below the position of the ophthalmic artery (**A**). In addition, the center of the aneurysm orifice center (red arrow) was assumed to be the location of the aneurysm formation site (**B**).

FSI was adopted to investigate the effects of the interaction between blood flow and the structures such as blood vessel and dura mater affecting the blood flow. The location of the dura mater was determined using the location of the ophthalmic artery because it typically originates from the anteromedial surface of the ICA just distal to the dural ring. We assumed the location of the dura mater to be just proximal to the origin of the ophthalmic artery^18^. Figure 2A shows examples of the blood vessel model along with dura mater and the ophthalmic artery.

The strain was calculated by dividing the deformation by the initial length:

$$ \varepsilon \,\, = \,\,\frac{\vartriangle L}{{L_{0} }} $$

where $\varepsilon$, $\vartriangle L$, and $L_{0}$ are strain, deformation, and initial length, respectively. Therefore, higher strain indicated that the blood vessels were more severely deformed. Also, the stress was calculated by dividing the force by the cross-sectional area:

$$ \sigma = \frac{F}{A}\, $$

where $\upsigma$, F, and A are stress, force, and cross-sectional area, respectively. Finally, the relationship between stress and strain can be easily explained by the following simple equation in terms of solid mechanics:

$$ \sigma \,\, = \,\,E \cdot \varepsilon \, $$

where E is Young’s modulus. The force obtained from CFD is transferred to the structural analysis in FSI to clarify the deformation of the blood vessel^19^. Young’s modulus, a mechanical property of the blood vessel, must be used to calculate its deformation. In this paper, the term “strain” refers to the equivalent strain, a representative scalar quantity which describes the state of strains in each direction. A detail general procedure of finite element analysis is illustrated in Supplementary Figure S1.

In order to improve the efficiency of the structural simulation, a four-node shell element with a size of 0.1 mm was used for modeling the dura mater and the blood vessel. The number of shell elements was 160,000–170,000 for both the blood vessel and dura mater. Since the dura mater is firmly fixed to the blood vessel, the interface between the dura mater and the blood vessel was assumed to be bonded. The material properties of the dura mater and the blood vessel were simplified to the linear property. The Young’s moduli of the dura mater and the blood vessel were assumed to be 50 MPa and 1.6 MPa, respectively^20,21^. In addition, Poisson’s ratio and density of the dura mater and the blood vessel were set to 0.49 and 1000 kg/m^3^_,_ respectively. Also, wall thicknesses of the dura mater and the blood vessel were defined from experimental data as 0.68 mm and 0.5 mm, respectively^20,21^. Additionally, all degrees of freedoms in the inlet, outlet, and edge of the dura mater were constrained as fixed. The values of WSS and strain were extracted at peak systole.

### Determination of the coordinates of the aneurysm formation site

In this study, we investigated the distance between the location of the aneurysm formation site and the locations of the results, *i.e.* high WSS, and high strain during quantitative comparison. Therefore, defining the coordinates of the aneurysm formation site was necessary. The point of the aneurysm formation site was assumed to be the center of the aneurysm ostium and the coordinates of the aneurysm formation site were calculated by overlapping models with and without aneurysms (Fig. 2B).

### Statistical analysis

Two independent t-tests were used to compare the distance between the aneurysm formation site and the locations of high WSS and high strain. Statistical significance was defined as a *p *value < 0.05. The distances were calculated using the following equation:

$$ {\text{The distance = }}\left| {\sqrt {\left( {x_{i} \, - \,x_{r} } \right)^{2} \, + \,\,\left( {y_{i} \, - \,y_{r} } \right)^{2} \, + \,\,\left( {z_{i} \, - \,z_{r} } \right)^{2} } } \right| $$

where ${x}_{i}$, ${y}_{i}$, and ${z}_{i}$ indicate the coordinate of the aneurysm initiation site and ${x}_{r}$, ${y}_{r}$, and ${z}_{r}$ indicate the coordinate of the results such as high WSS, and high strain. All statistical analyses were perform using IBM SPSS Statistics (version 24.0; IBM Corp., Armonk, NY, USA).

### Ethical approval

All procedures performed in this study involving human participants were in accordance with the ethical standards of the institutional and/or national research committee and with the 1964 Helsinki declaration and its later amendments or comparable ethical standards.

### Institutional review board

Catholic Kwandong University, International St. Mary’s Hospital, Institutional Review Board.

### Informed consent

Name of committee that waived the informed consent for the study protocol is Catholic Kwandong University, International St. Mary’s Hospital, Institutional Review Board.

## Results

### Superiority of the strain parameter

**Figure 3:**
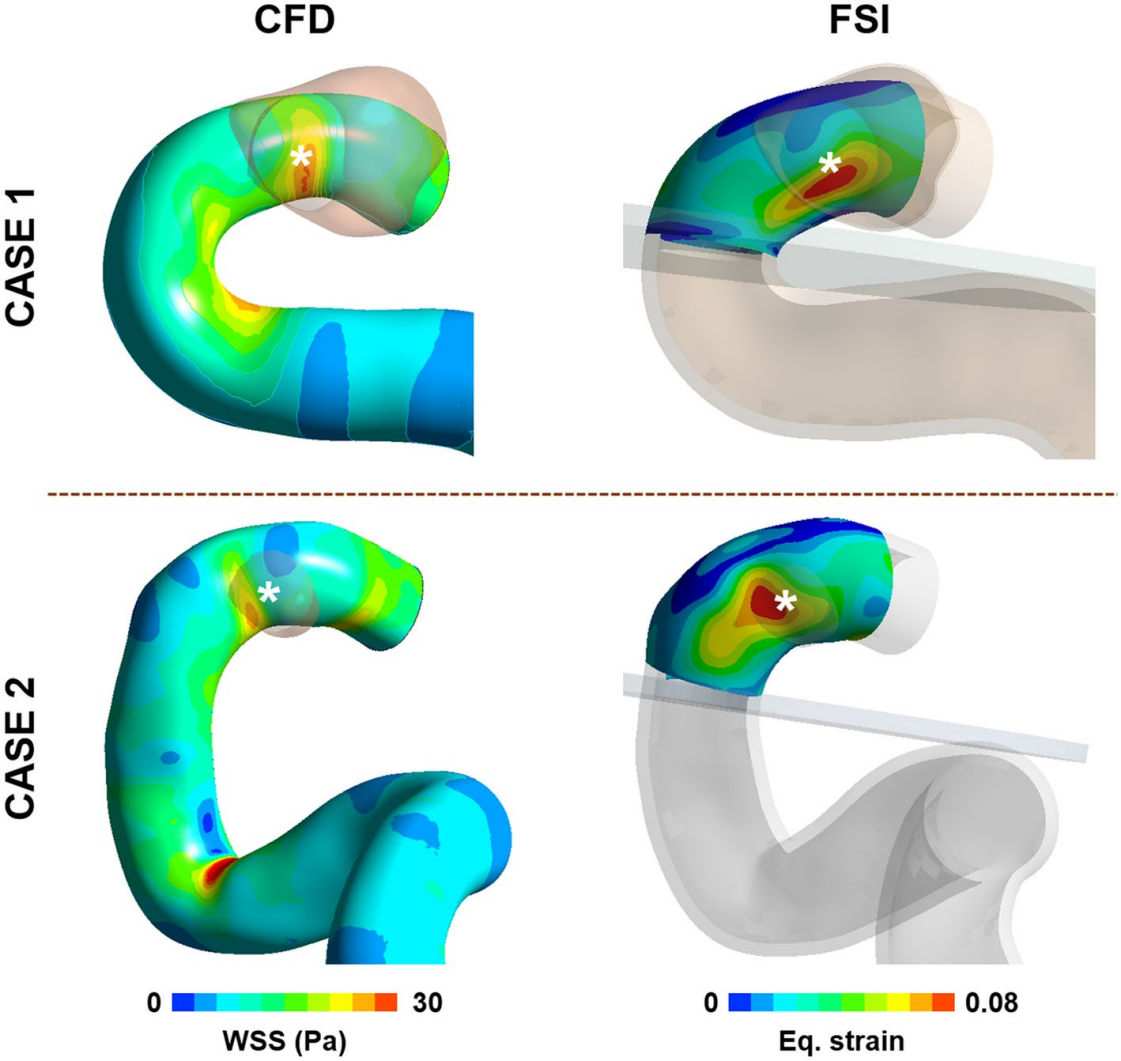
Examples which have high correlation between the location of the aneurysm formation site and the locations of the high WSS and high strain. The results of WSS calculated from CFD and strain calculated from FSI analysis were extracted at the systolic time for comparison of the location of high WSS and strain. A white star (*) indicates the location of aneurysm formation site.

**Figure 4:**
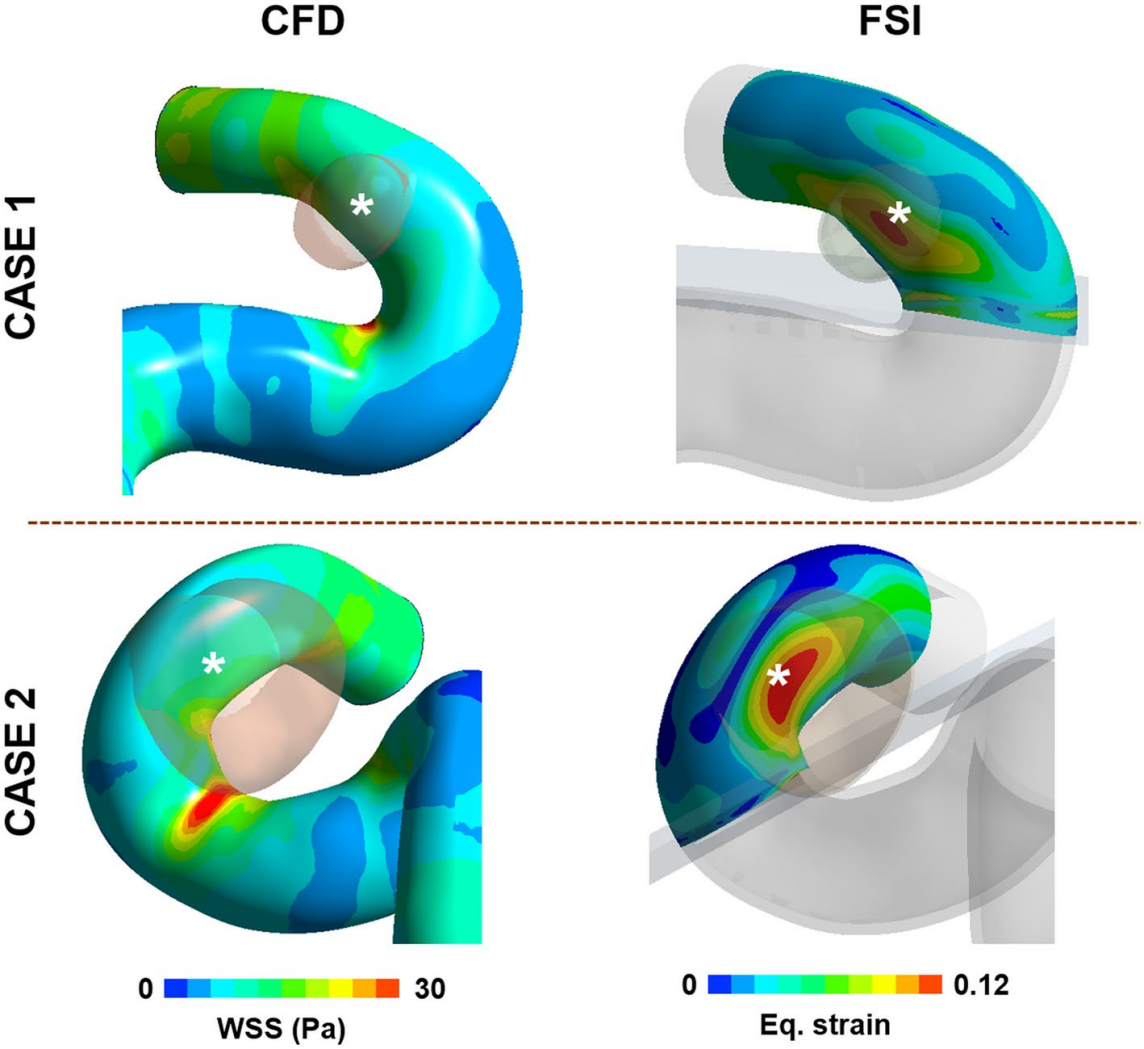
Examples where location of the aneurysm formation site is clear in correlation with high strain but not with high WSS. The location of high WSS and high strain was extracted from results of CFD and FSI analysis at the systolic time. A white star (*) indicates the location of aneurysm formation site.

In some cases, the locations of the aneurysm formation site were well matched to the locations of both high WSS in CFD analysis and high strain in FSI analysis (Fig. 3). However, in other cases, the location of the high strain calculated from FSI analysis was in good agreement with the location of aneurysm formation, but the location of high WSS calculated in CFD was not (Fig. 4).

**Figure 5:**
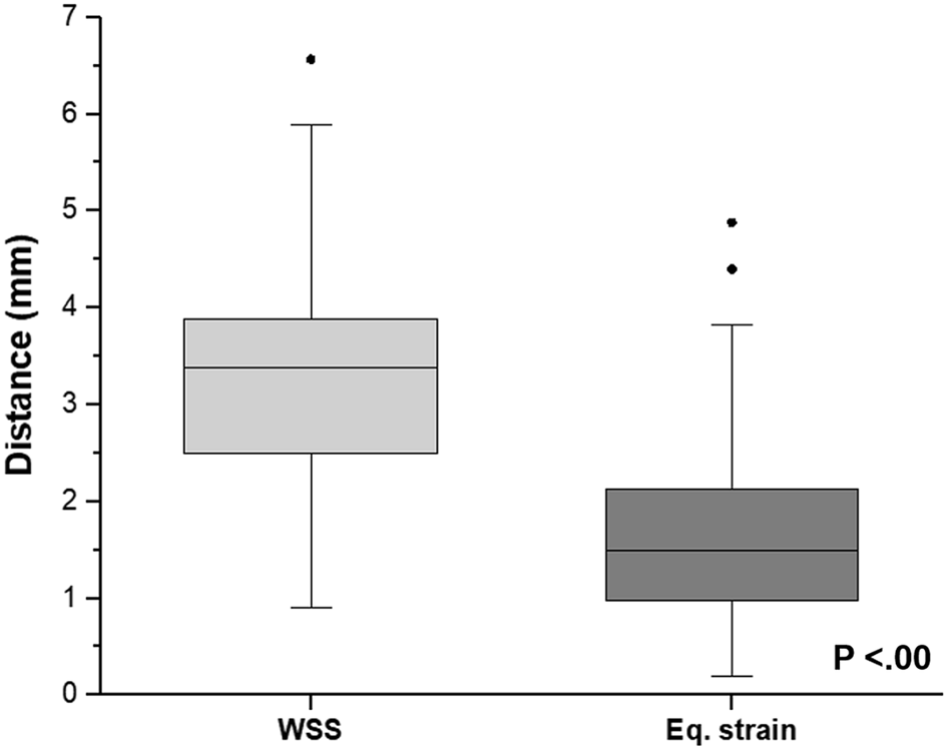
The box plot of the distance between the aneurysm formation site and the high WSS or high strain. The lower and upper borders represent the 25th and 75th percentiles, respectively, while the central bar indicates the median and the dots are outliers defined by values that are greater than the 90th or less than the 10th percentiles.

The average distance between the location of aneurysm formation and the location of the high WSS was 3.33 mm ($\pm$ 1.18 mm). On the other hand, the average distance to the point of high strain (1.74 mm $\pm$ 1.04 mm) was smaller than that to the high WSS. Figure 5 demonstrated that there was a statistical difference between the distance from the location of aneurysm formation to the high WSS and that to the location of high strain.

### The effects of dura mater

**Figure 6:**
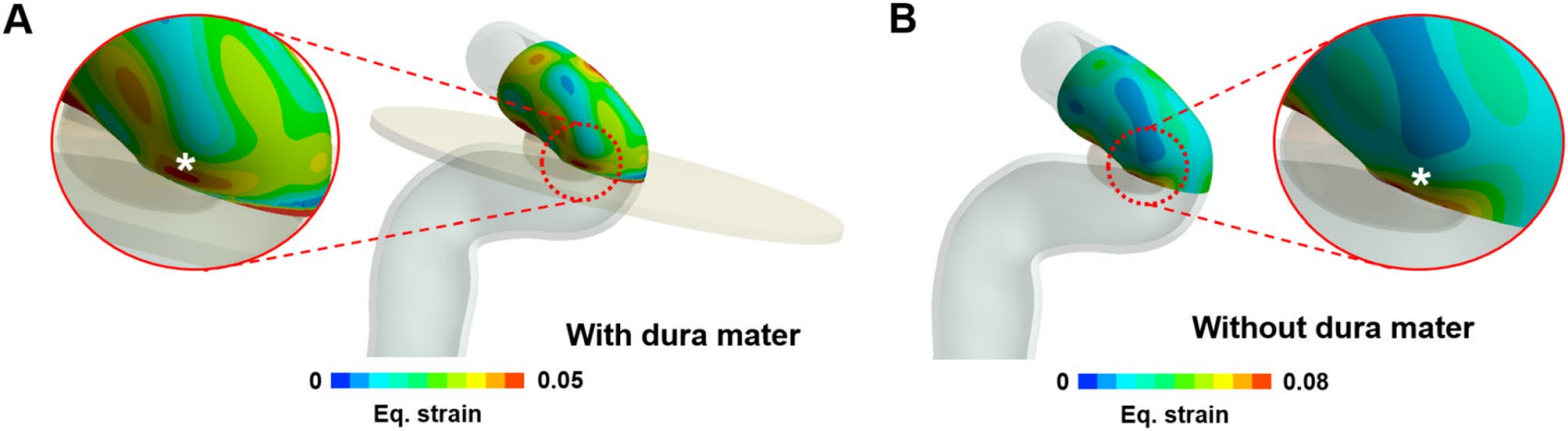
Example of strain distribution change depending on the presence of the dura mater. The strain distribution when the dura mater was considered (**A**). The strain distribution when the dura mater was not employed in FSI analysis (**B**). A white star (*) indicates the location of aneurysm formation site. The strain was extracted at the systolic time.

Figure 6 presents findings of a difference in strain distribution depending on the presence or absence of dura mater. The correlation between the locations of high strain and aneurysm formation was clear when the dura mater is considered (Fig. 6A). In contrast, without dura mater, it was relatively more difficult to specify the correlation between them (Fig. 6B). The FSI results with and without dura mater for the additional cases are shown in the Supplementary Figure S2.

**Figure 7:**
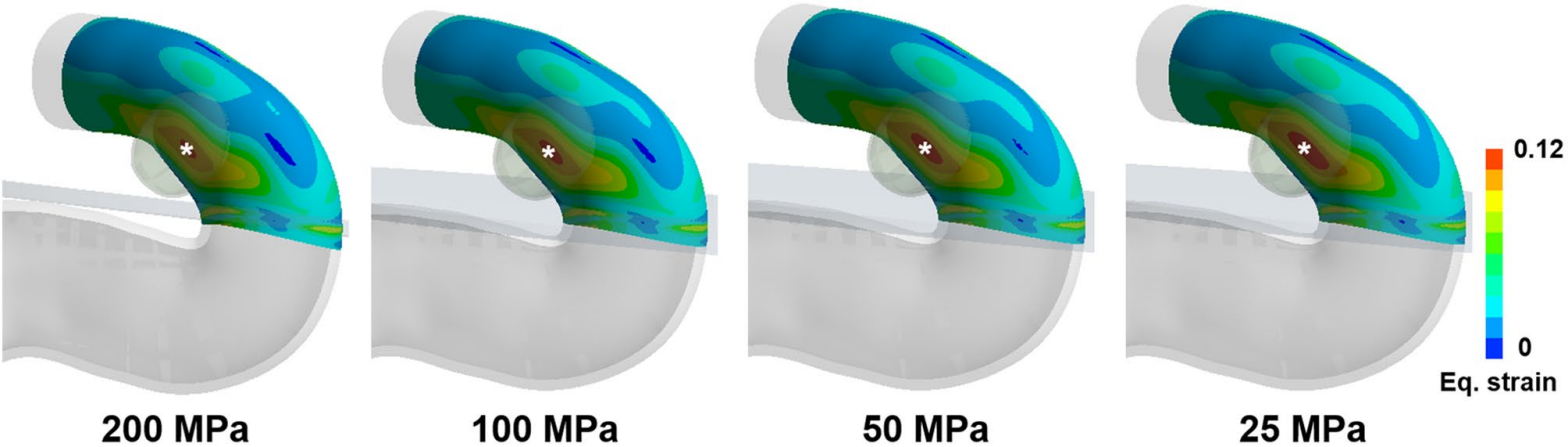
Example of strain contour change depending on Young’s modulus of the dura mater change. There is a difference in strain contour for various Young’s moduli of the dura mater. When Young’s modulus of the dura mater is 200 MPa, 100 MPa, 50 MPa, and 25 MPa, the max strain is 0.111, 0.112, 0.114, and 0.116, respectively. A white star (*) indicates the location of aneurysm formation site. The strain was extracted at the systolic time.

Moreover, the Young’s modulus of the dura mater affects the strain in the aneurysm formation site. As the Young’s modulus of the dura mater decreased, the value of strain in the aneurysm formation site was similarly increased. Meanwhile, the area with the high strain was increased as the Young’s modulus of the dura mater decreased (Fig. 7). It was confirmed that this trend was the same in other additional cases (Supplementary Figure S3).

## Discussion

FSI analysis is the simulation considering flow and structure together, whereas CFD analysis is the simulation considering only flow (e.g., blood flow). Naturally, blood vessels and blood flow through the blood vessels exhibit an interaction that affects both elements^22^. Therefore, the utilization of FSI analysis is more important in analyzing the nature of actual ongoing phenomena^23^.

In previous studies, WSS has been known as one of the most important hemodynamic factors^5,8–10^, while pressure as a hemodynamic factor appeared to be less important. In particular, many researchers seem to agree on the notion that elevated WSS and the location of aneurysm formation achieve a high positive correlation. However, most previous hemodynamic studies of aneurysm formation have been conducted mainly through CFD analysis. Therefore, we planned to compare the influence of strain on aneurysm formation based on FSI analysis with that of WSS based on the CFD analysis.

### Quantitative comparison of WSS and strain

Even though the aneurysm formation site could be predicted with the WSS only from CFD, the results of the strain calculated during FSI analysis suggest a more intuitive correlation between the strain and the aneurysm formation site than between the WSS and the aneurysm formation site (Fig. 5). Many studies have suggested that the mechanical stretch in the vessel wall is a major reason for aneurysm formation^5,10^. The strain is a mechanical quantity that directly represents how much the vessel wall is stretched. This may be one of the reasons for the high correlation between strain and aneurysm formation.

### Effects of the dura mater

In the FSI analysis of paraclinoid aneurysm formation, it was confirmed that strain distribution appeared differently depending on the presence or absence of the dura mater. Like the actual structure, when we included the dura mater surrounding the blood vessels in the analysis, we could see that the high strain region and the location of aneurysm formation were more clearly matched. This suggests that the dura mater could affect the formation of paraclinoid aneurysms.

In addition, as the material property of the dura decreased, we observed a slight increase in the strain value as well as the area with high strain in the same vessel area. This might be correlated with the increased possibility of aneurysm formation among older patients because their dura mater has a decreased material property^20^.

### The outcomes of FSI analysis in various types of paraclinoid aneurysms

**Figure 8:**
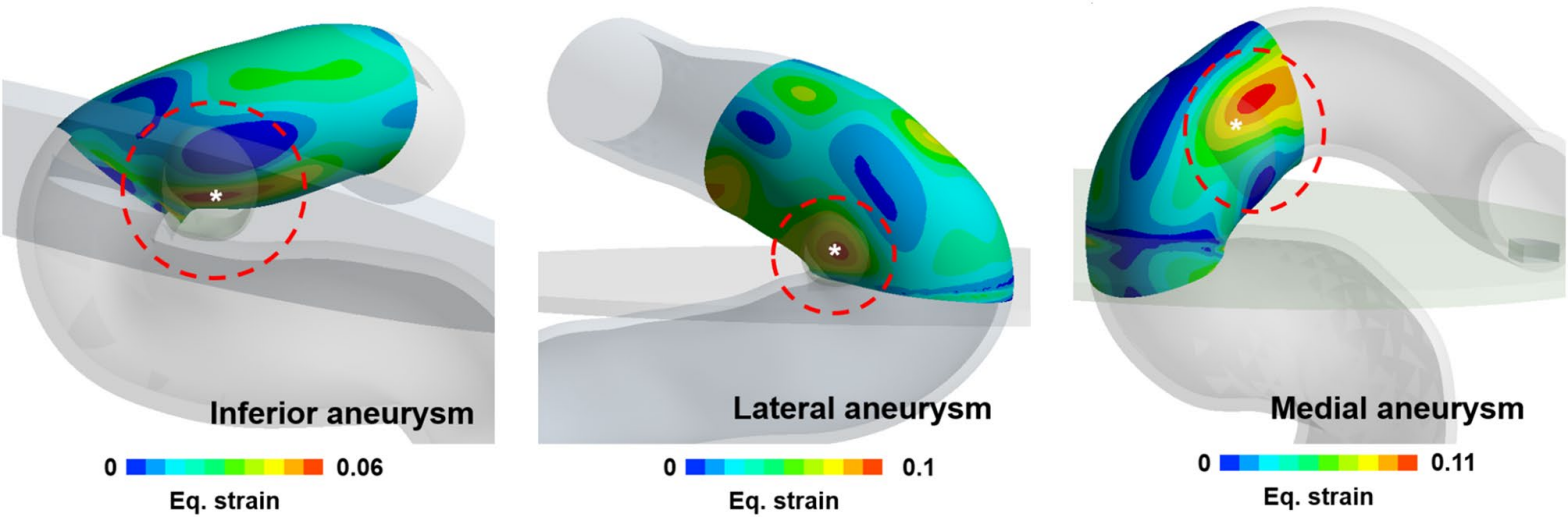
Examples of FSI results by paraclinoid aneurysm types. The strain distributions for paraclinoid models which have different aneurysm types were calculated using FSI analysis. A white star (*) indicates the location of aneurysm formation site. The strain was extracted at the systolic time.

There are many types of paraclinoid aneurysms^24^. To date, medical research has not clearly explained the cause of an aneurysm occurrence in several directions. However, the direction of a paraclinoid aneurysm can be explained by the FSI analysis, as the location of aneurysm formation varies depending on where the high strain appears (Fig. 8). In other words, it may be considered that the formation of paraclinoid aneurysms at different locations depends on the shape of the blood vessels such as curvature and twist of vessels, and this could be observed using FSI.

## Limitations

This study is limited by its inherent accuracies. The material property of blood vessels and inflow profiles used in this study were not patient-specific and taken from a cohort of healthy patients. Blood flow was assumed Newtonian. Another limitation was that the precise location of the dura mater attached to the blood vessels was also assumed. We set the location with the advice of an experienced neurosurgeon based on magnetic resonance images, but there is a possibility that we may have not chosen the correct position. Additionally, we assumed the aneurysm formation initiates at the center of the aneurysm orifice; however, this has yet to be clearly demonstrated and could be a limitation of this study.

## Conclusions

In this study, using a more realistic FSI analysis approach, we were able to identify the importance of strain among the hemodynamic parameters. Considering strain in the future could help predict the formation of aneurysms and elucidate appropriate treatments.

## Supplementary Information

Supplementary Information

## References

[1] Jamous MA, Nagahiro S, Kitazato KT, Satoh K, Satomi J (2005). Vascular corrosion casts mirroring early morphological changes that lead to the formation of saccular cerebral aneurysm: an experimental study in rats. J. Neurosurg..

[2] Meng H, Tutino VM, Xiang J, Siddiqui A (2014). High WSS or low WSS? Complex interactions of hemodynamics with intracranial aneurysm initiation, growth, and rupture: toward a unifying hypothesis. AJNR Am. J. Neuroradiol..

[3] Can A, Du R (2016). Association of hemodynamic factors with intracranial aneurysm formation and rupture: systematic review and meta-analysis. Neurosurgery.

[4] Etminan N, Rinkel GJ (2016). Unruptured intracranial aneurysms: development, rupture and preventive management. Nat. Rev. Neurol..

[5] Frosen J, Cebral J, Robertson AM, Aoki T (2019). Flow-induced, inflammation-mediated arterial wall remodeling in the formation and progression of intracranial aneurysms. Neurosurg. Focus.

[6] Murayama Y, Fujimura S, Suzuki T, Takao H (2019). Computational fluid dynamics as a risk assessment tool for aneurysm rupture. Neurosurg. Focus.

[7] Lauric A (2019). Proximal parent vessel tapering is associated with aneurysm at the middle cerebral artery bifurcation. Neurosurgery.

[8] Lauric A, Hippelheuser J, Safain MG, Malek AM (2014). Curvature effect on hemodynamic conditions at the inner bend of the carotid siphon and its relation to aneurysm formation. J. Biomech..

[9] Chen H (2013). Investigating the influence of haemodynamic stimuli on intracranial aneurysm inception. Ann. Biomed. Eng..

[10] Soldozy S (2019). The biophysical role of hemodynamics in the pathogenesis of cerebral aneurysm formation and rupture. Neurosurg. Focus.

[11] Gao L (2008). Nascent aneurysm formation at the basilar terminus induced by hemodynamics. Stroke.

[12] Frosen J (2016). Flow dynamics of aneurysm growth and rupture: challenges for the development of computational flow dynamics as a diagnostic tool to detect rupture-prone aneurysms. Acta Neurochir. Suppl..

[13] Sanchez M (2014). Intracranial aneurysmal pulsatility as a new individual criterion for rupture risk evaluation: biomechanical and numeric approach (IRRAs Project). AJNR Am. J. Neuroradiol..

[14] Cho KC, Choi JH, Oh JH, Kim YB (2018). Prediction of thin-walled areas of unruptured cerebral aneurysms through comparison of normalized hemodynamic parameters and intraoperative images. Biomed. Res. Int..

[15] Hua Y, Oh JH, Kim YB (2015). Influence of parent artery segmentation and boundary conditions on hemodynamic characteristics of intracranial aneurysms. Yonsei Med. J..

[16] Kono K, Fujimoto T, Shintani A, Terada T (2012). Hemodynamic characteristics at the rupture site of cerebral aneurysms: a case study. Neurosurgery.

[17] Reymond P, Merenda F, Perren F, Rufenacht D, Stergiopulos N (2009). Validation of a one-dimensional model of the systemic arterial tree. Am. J. Physiol. Heart Circ. Physiol..

[18] Horiuchi T (2009). Relationship between the ophthalmic artery and the dural ring of the internal carotid artery. Clinical article. J. Neurosurg..

[19] Beckert A (2000). Coupling fluid (CFD) and structural (FE) models using finite interpolation elements. Aerosp. Sci. Technol.

[20] Zwirner J, Scholze M, Waddell JN, Ondruschka B, Hammer N (2019). Mechanical properties of human dura mater in tension—an analysis at an age range of 2 to 94 years. Sci Rep.

[21] Alastruey J, Parker KH, Peiro J, Byrd SM, Sherwin SJ (2007). Modelling the circle of Willis to assess the effects of anatomical variations and occlusions on cerebral flows. J. Biomech..

[22] Torii R, Oshima M, Kobayashi T, Takagi K, Tezduyar TE (2006). Fluid–structure interaction modeling of aneurysmal conditions with high and normal blood pressures. Comput. Mech..

[23] Torii R, Oshima M, Kobayashi T, Takagi K, Tezduyar TE (2009). Fluid–structure interaction modeling of blood flow and cerebral aneurysm: Significance of artery and aneurysm shapes. Comput. Methods Appl. Mech. Eng..

[24] Chen L (2008). Usefulness of a simplified management scheme for paraclinoid aneurysms based on a modified classification. Cerebrovasc. Dis..

