# Supplementary material for: The quantitative comparison between high wall shear stress and high strain in the formation of paraclinoid aneurysms
Source: Sci Rep. 2021 Apr 12;11:7947. doi: 10.1038/s41598-021-87126-w (PMC8041878; doi:10.1038/s41598-021-87126-w)
Supplement: Supplementary file 1 — Supplementary Information [file 41598_2021_87126_MOESM1_ESM.docx]

**Supplementary Information**

**The Quantitative Comparison Between High Wall Shear Stress and High Strain in the Formation of Paraclinoid Aneurysms**

*Jung-Jae Kim, MD^1^, *Hyeondong Yang, MS^2^, Yong Bae Kim, MD, PhD^3^, Je Hoon Oh, PhD^2^, Kwang-Chun Cho, MD^4^

^1^Department of Neurosurgery, College of Medicine, Ewha Womans University, Ewha Womans University Seoul Hospital, Seoul, Korea

^2^Department of Mechanical Engineering and BK21 FOUR ERICA-ACE Center, Hanyang University, Ansan, Gyeonggi-do, Korea

^3^Department of Neurosurgery, College of Medicine, Yonsei University, Severance Hospital, Seoul, Korea

^4^Department of Neurosurgery, College of Medicine, Catholic Kwandong University, International St. Mary’s Hospital, Incheon, Korea

*JJ Kim and HD Yang contributed equally to this work as co–first authors.

JH Oh and KC Cho contributed equally to this work as co–corresponding authors.


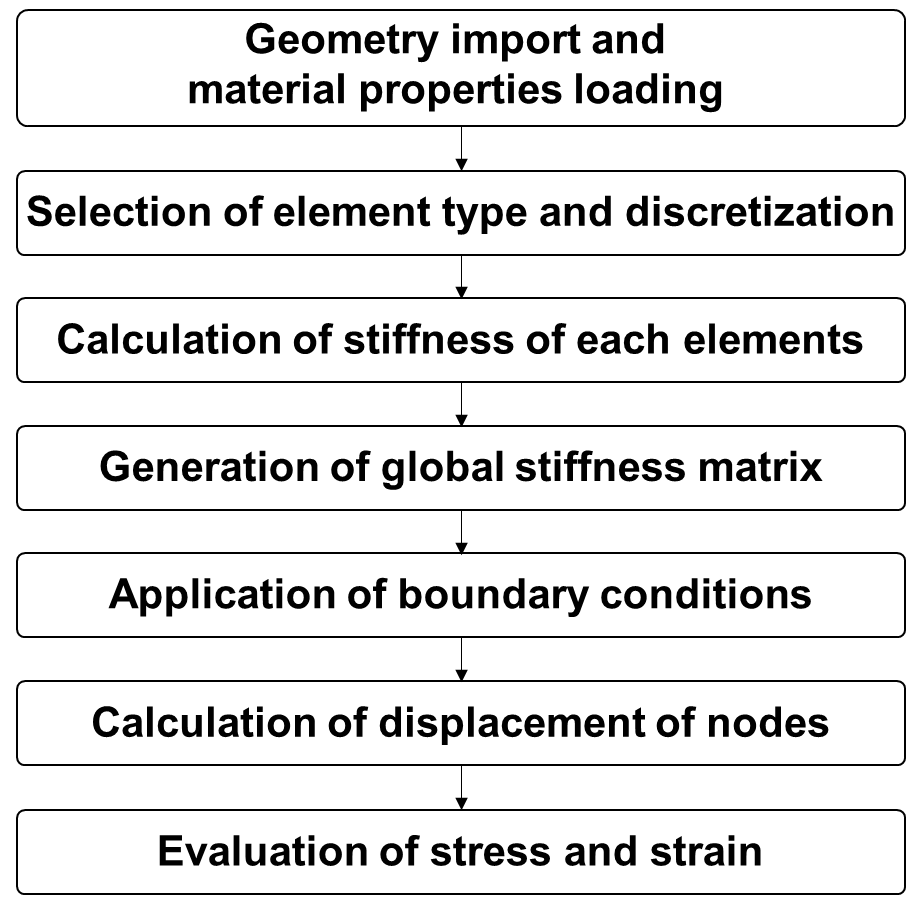


**Supplementary Figure S1.** General procedure of finite element analysis

Generally, there are several steps to calculate stress and strain using finite element method. (Figure S1) First step is element division and node numbering. To solve the problem using finite element method, the geometry is discretized by elements. In this step, the properties of element such as a type, a size are determined. Second, a shape function of element is determined. In order to assume the displacement of node in element, various shape functions are utilized. Third, a relationship between strain and displacement and a stress and strain relation with the displacement are determined. The material properties such as Young’s modulus are applied at this step. Fourth, stiffness matrix and load vector of elements are constructed. Fifth, a global stiffness matrix and load vectors are assembled. sixth, a boundary conditions are applied to the constructed matrix. seventh, the displacements of nodes are calculated using numerical methods. Finally, stress and strain were evaluated with the calculated displacement.


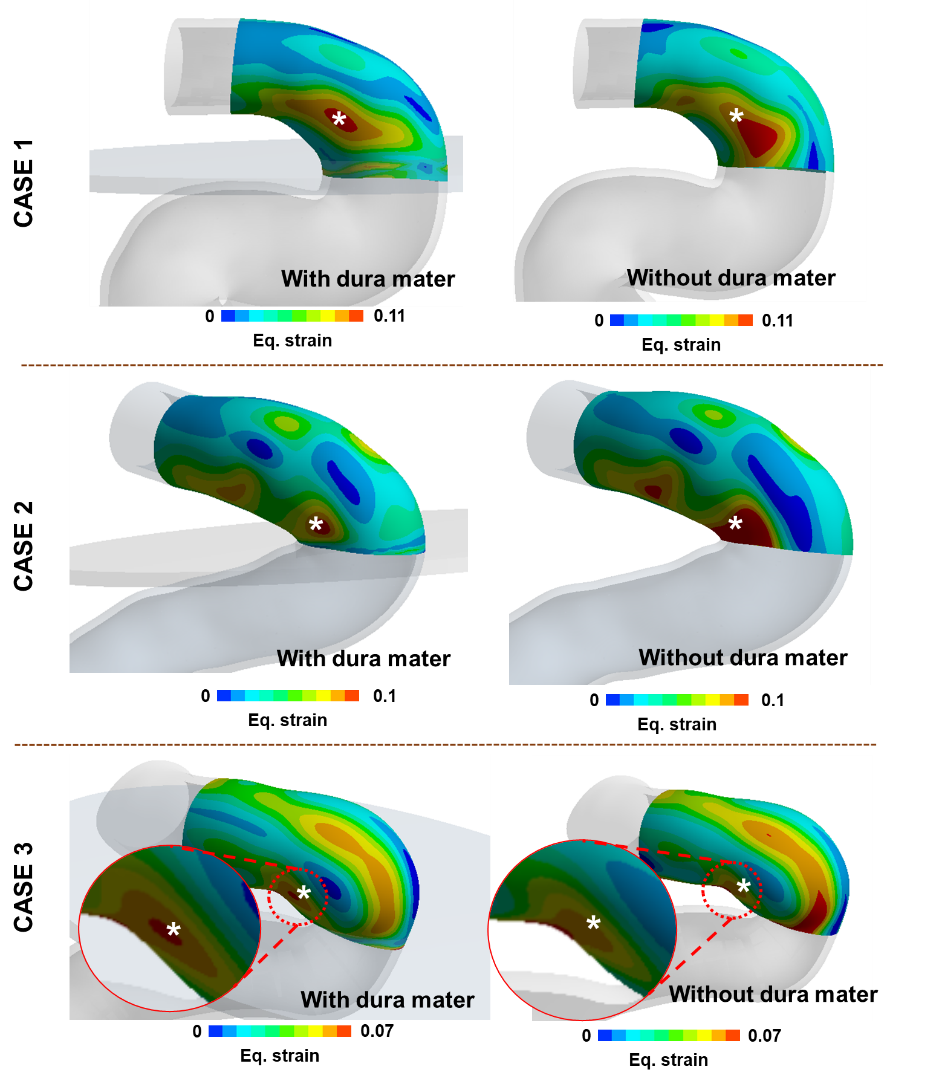


**Supplementary Figure S2****.** The difference of strain distribution calculated in FSI analysis depending on the employment of the dura mater for additional cases. A white star (*) indicates the location of aneurysm formation site. The strain was extracted at the systolic time.


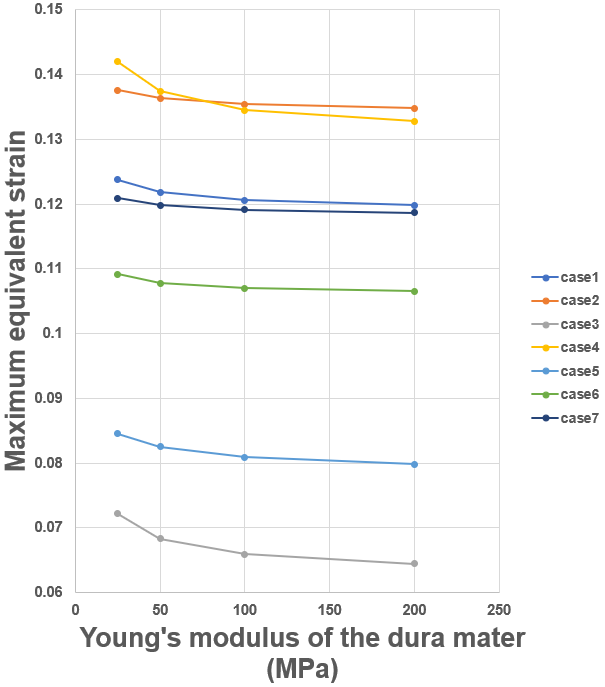


**Supplementary Figure S3.** Graph of the changes in maximum strain values depending on Young’s modulus of the dura mater change for additional cases. The maximum strain results of varying Young’s modulus were calculated for additional cases using FSI analysis. The strain was extracted at the systolic time.
